# Predictivity of fatty liver index for non-alcoholic fatty liver disease in lean females with polycystic ovary syndrome

**DOI:** 10.4314/ahs.v22i1.75

**Published:** 2022-03

**Authors:** Didem Arıkan, Attila Önmez, Erson Aksu, Nicel Taşdemir

**Affiliations:** 1 Department of Obstetrics and Gynecology, Derince Research and Training Hospital, Kocaeli, Turkey; 2 Department of Internal Medicine, Düzce University Faculty of Medicine, Düzce, Turkey; 3 Department of Obstetrics and Gynecology, Çorlu Vatan Hospital, Tekirdağ, Turkey; 4 Department of Obstetrics and Gynecology, Acibadem Hospitals Group, Eskişehir, Turkey

**Keywords:** Polycystic ovary syndrome, non-alcoholic fatty liver disease, fatty liver index

## Abstract

**Background:**

Fatty liver index (FLI) is a simple tool used to predict non-alcoholic fatty liver disease (NAFLD). The role of FLI in polycystic ovary syndrome (PCOS) for the prediction of NAFLD has not been elucidated.

**Methods:**

This case-control study was from January 2014 to January 2016. Anthropometric measurements, biochemical testing, and abdominal ultrasonography were performed in 83 premenopausal otherwise healthy women with PCOS and 58 controls. NAFLD was diagnosed by ultrasound. The predictivity of FLI for NAFLD in lean and overweight/obese females with PCOS was analyzed.

**Results:**

The γ-glutamyl transferase levels were significantly higher in the females with PCOS than in the controls (p = 0.001). In women with PCOS, FLI was significantly higher in females with NAFLD comparing to those without NAFLD (47.1 ± 33.6 vs. 16.9 ± 21.6; p = 0.001). For the PCOS group, Body Mass Index had the strongest relationship with FLI (p < 0.05, r = 0.908). FLI < 30 was calculated for all the lean females. The lean females with PCOS had a significantly higher rate of NAFLD (27.5% vs. 8.8%; p = 0.041) than lean controls.

**Conclusion:**

An FLI < 30 was not sufficient to rule out NAFLD in the lean PCOS patients.

## Introduction

Polycystic ovary syndrome (PCOS) is seen in approximately 6.5% of premenopausal females, and it is one of the most common endocrinopathies associated with reproductive and metabolic abnormalities^1^. Metabolic abnormalities, such as obesity, impaired glucose tolerance, insulin resistance, dyslipidemia, chronic inflammation, and increased oxidative stress, are often present in patients with PCOS^2^.

Non-alcoholic fatty liver disease (NAFLD) is defined as steatosis without alcoholism. The term NAFLD refers to conditions ranging from simple hepatic steatosis to endstage liver disease. Obesity, hyperglycaemia, hyperinsulinemia, hypertriglyceridemia, and systolic hypertension are associated with NAFLD^3^. The condition shows characteristics of metabolic syndrom^4^. The metabolic results of PCOS and the etiological factors of NAFLD are very similar. The risk for NAFLD is higher in females with PCOS than in females without PCOS^5^.

Approximately 75% of obese individuals develop NAFLD, compared to 3.5%–24% of the general population^6^. Of the females with PCOS, 50% are overweight or obese^7^. Some studies have indicated that NAFLD is the hepatic outcome of PCOS. Others have suggested that metabolic syndrome manifests as NAFLD in the liver, and PCOS in the ovaries^8^–^11^. An association between high body mass index (BMI) and the development of NAFLD in PCOS patients has been reported^7^, ^12^–^14^. Gambarin-Gelwan et al.^13^ suggested that obesity was a major determinant for NAFLD in patients with PCOS. Interestingly, the study found ultrasonographic evidence of hepatic steatosis in 39% of lean women with PCOS a high rate despite exclusion of the effects of obesity.

The gold standard for the diagnosis of NAFLD is liver biopsy. Liver ultrasonography (USG) has been shown to have a high sensitivity and specificity for the diagnosis of NAFLD in cases with hepatic steatosis^15^. USG has also been described as the only technique that, for ethical and logistic reasons, can be used to diagnose fatty liver in the general population^16^. In a meta-analysis, ultrasound was compared with histological diagnosis and was found to be reliable and accurate^17^.

Bedogni et al.^18^. demonstrated that the fatty liver index (FLI), an algorithm based on BMI, waist circumference, triglyceride levels, and gamma (γ)-glutamyl transferase (GGT) is another non-invasive method for detecting NAFLD in the general population. An FLI < 30 rules fatty liver out, and an FLI ≥ 60 rules it in^18^, ^19^. Lerchbaum et al.^1^ indicated that FLI > 60 was a common finding in obese women with PCOS and that it was linked to metabolic syndrome; however, the study did not investigate the relationship between diagnosed NAFLD and FLI in females with PCOS.

The present study used USG to investigate the predictivity of FLI for NAFLD diagnoses in lean and overweight/obese PCOS patients. In addition, comparisons of BMI, waist circumference, age, GGT, and triglyceride levels between 1- controls and females with PCOS; 2- females with PCOS, with and without NAFLD were done.

## Materials and methods

The study population was composed of females admitted at the Namık Kemal University Faculty of Medicine Department of Obstetrics and Gynecology from January 2014 to January 2016. Premenopausal volunteer women, aged between 18–47 years were included. We excluded those who had received treatment for PCOS within the previous six months, pregnant women, those who consumed more than 20 g alcohol daily, those who had used oral contraceptives within the previous three months, and those with any of the following diagnoses: hypothyroidism, hyperthyroidism, hyperprolactinemia, diabetes mellitus, Cushing syndrome, congenital adrenal hyperplasia, androgen-secreting ovarian or adrenal tumor, chronic viral hepatitis, hemochromatosis and autoimmune hepatitis, neoplastic, metabolic, or cardiovascular diseases, and a history of chronic diseases. The control group was composed of healthy females who had undergone abdominal and/or liver ultrasonography performed by the same radiologist for any reason within the last six months of the study period. All relevant medical records of the patients and controls were retrospectively evaluated.

The study protocol was approved by the Faculty of Medicine Ethical Committee. All of the patients provided informed consent. The patient group comprised 83 newly diagnosed PCOS patients, and the control group had 58 healthy females. The Rotterdam consensus criteria (2003) were used to diagnose PCOS (20): 1- Oligoovulation (less than 6 menses per year) or anovulation (less than 2 menses per year). 2- Proof of hyperandrogenism by either laboratory studies (measurement of androgen levels) or clinical findings (hirsutism [mFG score >8], acne, seborrhea, hoarse voice, weight increase, androgenic alopecia). 3- Polycystic ovaries in ultrasonography imaging (≥12 follicles (2–9 mm diameter) and/or increased ovary volume (>10 ml)).

### Study protocol

The obstetric and gynecologic histories, menstrual cycles, reproductive histories, diseases, and medications for all of the participants were assessed. Anthropometric measurements were performed, and the BMI (kg/m^2^) was calculated. The participants were classified by their nutritional status using BMI. A BMI ≤ 25 was defined as lean patients, and a BMI > 25 was defined as overweight/obese. Following a physical examination, USG was performed transvaginally on participants who had approved this approach, and transabdominally. Five mls of venous blood was withdrawn from all participants between the 3^rd^ and 5^th^ days of their menstruation. Blood samples were drawn in the morning, between 8.00 and 9.30, after overnight fasting. The levels of glucose, total cholesterol, high density lipoprotein, low density lipoprotein, triglyceride and gamma glutamyl transferase (GGT) were measured. Oral glucose tolerance test (75 g) and HbA1c levels are measured to exclude diabetes mellitus.

The FLI values for the patients was calculated by the following formula:

FLI = (e 0.953*loge (triglycerides) + 0.139*BMI + 0.718*loge (GGT) + 0.053*waist circumference − 15.745) / (1 + e 0.953*loge (triglycerides) + 0.139*BMI + 0.718*loge (GGT) + 0.053*waist circumference −15.745) * 100 (18).

The absence of hepatic steatosis was considered as a homogenous texture, with fine level echoes and isoechoic compared to the renal cortex and adequate visualization of the hepatic vessels and diaphragm with USG B-mode imaging. Grade 1 hepatic steatosis was defined as the presence of bright echoes or increased hepatorenal contrast, Grade 2 hepatic steatosis as the presence of both bright echoes and increased hepatoreal contrast, as well as vessel blurring and Grade 3 steatosis, was considered to be present when in addition to the criteria for Grade 2, there was evidence of posterior beam attenuation and non-visualization of the diaphragm.^15^, ^21^ Since patients with alcohol use were not included in the study, those with signs of hepatic steatosis on abdominal USG were diagnosed with NAFLD.

### Statistical analysis

All statistical analyses were performed with SPSS Statistics for Windows version 18.0 (SPSS Inc., Chicago, Illinois, USA). Parametric data were given as mean ± standard deviation (SD). Normality of distribution was tested with the Shapiro-Wilk test. The categorical variables were compared with the chi-square test. The Student's t-test was used to compare the normally distributed parametric variables, and the Mann Whitney U test was used to compare the parametric variables that did not show a normal distribution. Pearson correlation coefficient wass used to measure the relationship between variables. The value p < 0.05 was accepted for demonstrating statistical significance.

## Results

### Control group vs. PCOS group

There were no statistically significant differences in waist circumference, BMI, triglyceride levels, NAFLD presence, or FLI between the control group and the patients with PCOS (p=0.146, p=0.155, p=0.810, p=0.431, p=0.106; respectively). The ages in the PCOS group were lower than those in the control group, and the GGT activity was higher (p=0.002, p=0.001; respectively). The findings are presented in Table 1.

**Table 1:** 

Parameters	Control (n = 58)	PCOS (n = 83)	p
Age (year)	27.7 ± 6.2	24.7 ± 5.5	0.002**
GGT (U/L)	12.2 ± 6.9	15.7 ± 10	0.001*
Waist circumference (cm)	83.6 ± 13.1	87.2 ± 14.8	0.146**
BMI (kg/m^2^)	24.5 ± 4.7	25.8 ± 5.8	0.155**
Triglyceride (mg/dL)	107 ± 69.2	109.9 ± 68.8	0.810**
NAFLD Grade 1 Grade 2	22 (37.9%) 19 (32.8%) 3 (5.1%)	37 (44.6%) 32 (38.6%) 5 (6%)	0.431***
FLI	22.4 ± 26.8	30.3 ± 31.3	0.106*

Comparison of parameters in regard to patient and control groups

* Mann-Whitney U test

** Students t-test

*** Chi-square test

BMI: body mass index; FLI: fatty liver index; GGT: γ-glutamyl transferase; NAFLD: non-alcoholic fatty liver disease

According to the USG evaluations, 36 (62%) of the 58 controls did not have fatty liver, 19 (32.8%) were found to have grade 1 steatosis, and 3 (5.1%) had grade 2 steatosis. None of the controls was found to have grade 3 steatosis. Of the 83 patients diagnosed with PCOS, the USG indicated that 46 (55.4%) were found to have normal liver echogenicity but not fatty liver. Thirty-two (38.6%) patients were found to have grade 1 hepatic steatosis, and the remaining 5 (6%) were found to have grade 2 hepatic steatosis. None of the patients had grade 3 steatosis.

### Presence vs. absence of NAFLD in PCOS group

There were no statistically significant differences in age and GGT activity (p=0.416, p=0.229; respectively). Waist circumference (p=0.001), BMI (p=0.001), triglyceride levels (p=0.001), and FLI (p=0.001) were higher in the patients with NAFLD in the PCOS group than in the patients without NAFLD in the PCOS group. The results for the PCOS group with and without NAFLD are compared in Table 2.

**Table 2:** 

Parameters	NAFLD (-) (n = 46)	NAFLD (+) (n = 37)	p
Age (year)	25.7 ± 5.7	26.3 ± 6.4	0.416**
GGT (U/L)	14.1 ± 6.1	17.7 ± 13.2	0.229*
Waist Circumference (cm)	80.6 ± 12.8	95.3 ± 13.2	0.001**
BMI (kg/m^2^)	23.5 ± 4.5	28.7 ± 5.9	0.001**
Triglyceride (mg/dL)	86.5 ± 38.9	138.9 ± 85.6	0.001**
FLI	16.9 ± 21.6	47.1 ± 33.6	0.001*

Results and comparison of parameters according to the presence and absence of non-alcoholic fatty liver disease in polycystic ovary syndrome patients (n = 83).

* Mann-Whitney U test

** Students t-test

BMI: body mass index; FLI: fatty liver index, GGT: γ-glutamyl transferase; NAFLD: non-alcoholic fatty liver disease; NAFLD (+): NAFLD is diagnosed with USG; NAFLD (.) No NAFLD was found with USG; PCOS: polycystic ovary syndrome; USG: ultrasonography.

### Fatty liver index

For the entire study population, the FLI was significantly higher for the patients with NAFLD than for the patients without NAFLD (45.5 ± 32 vs. 13.8 ± 19.1; p = 0.001). In the PCOS group, the FLI was significantly higher for the patients with NAFLD than for the patients without NAFLD (47.1 ± 33.6 vs. 16.9 ± 21.6; p = 0.001) (Table 2).

For the controls (n = 58), the FLI values were < 30 for 42 participants (72.4%), 30–60 for 9 (15.5%) and > 60 for 7 (12%). In the PCOS group (n = 83), FLI < 30 was observed in 54 (65%) patients, FLI 30–60 in 12 (14.4%), and FLI > 60 in 17 (20.4%).

For the PCOS group, the Pearson correlation coefficient indicated that BMI (p < 0.05, r = 0.908), waist circumference (p < 0.05, r = 0.896), NAFLD (p < 0.05, r = 0.589), and age (p < 0.05, r = 0.180) were positively correlated with FLI. BMI had the strongest relationship with FLI.

### Body mass index

The PCOS and control groups were determined according to BMI. Of these subjects, 52% (n = 74) were lean, 48% (n = 67) were overweight or obese. Of the patients with NAFLD, 14 were lean, and 45 were overweight/obese. (Table 3)

**Table 3:** 

		NAFLD(+)	p*
Lean	PCOS	11 (27.5%)	0.041
	Control	3 (8.8%)	
Overweight / obese	PCOS	26 (60.5%)	0.118
	Control	19 (79.2%)	

Comparison of non-alcoholic fatty liver disease (NAFLD) presence between polycystic ovary syndrome and control groups in lean (body mass index [BMI ≤ 25]) participants and overweight/obese (body mass index > 25]) participants. All lean patients had a fatty liver index lower than 30.

* Chi-square test.

NAFLD (+): NAFLD is diagnosed with USG

### NAFLD (+): NAFLD is diagnosed with USG

**Figure 1:**
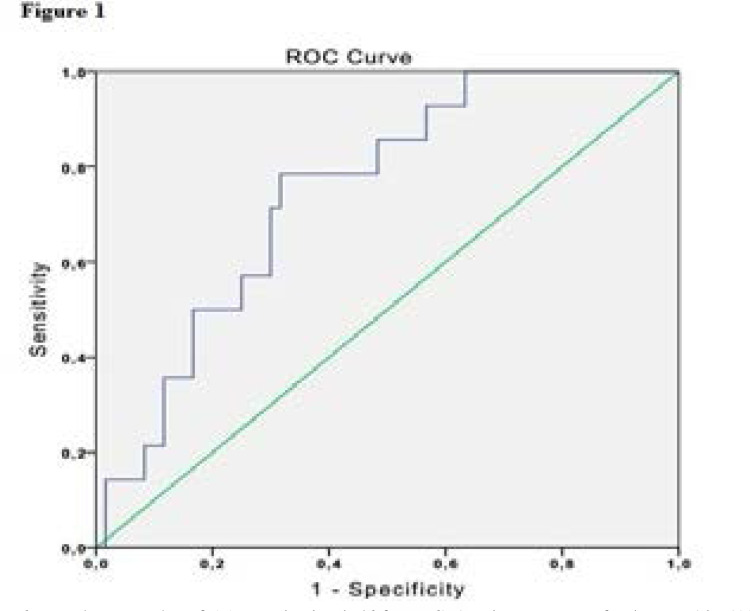
For lean PCOS patients and controls a FLI value of 5.68 was the threshold for predicting the prescence of NAFLD with 79% sensitivity and 68% specificity in the receiver-operating curve (ROC) (area under the curve = 0.748; CI = 0.623–0.872; p = 0.004). NAFLD: non-alcoholic fatty liver disease; PCOS: polycystic ovary syndrome; FLI: Fatty liver index.

Of the lean participants, NAFLD was present in 27.5% of the PCOS group but in only 8.8% of the control group. The difference between the groups was significant (p = 0.041) (Table 3). All the lean patients had a FLI < 30. Thus, 5.68 was calculated as a new cut-off value for FLI for the lean PCOS and control patients, with 79% sensitivity and 68% specificity in the receiver-operating curve (area under the curve = 0.748; CI = 0.623–0.872; p = 0.004) (Figure 1).

Regarding the overweight/obese patients, there was no difference between the PCOS and the control groups regarding NAFLD (p = 0.118) (Table 3).

## Discussion

NAFLD has been associated with several chronic diseases. Patients with NAFLD have higher mortality rates than the general population^22^, ^23^. Surveillance is warranted for patients with PCOS who are at risk for NAFLD.

The GGT activity was significantly higher in the PCOS group than in the healthy controls. This supports the results of Ozturk et al.^24^, who found higher GGT activity in women with PCOS than in the controls, independent of BMI.

In the general population, an age higher than 40 has been found to be a risk factor for NAFLD^16^, ^25^. In our study the difference in the ages of the women in the control and the PCOS groups was not great enough to have an effect on the development of NAFLD.

In the present study, there was no significant difference between the PCOS and the control groups regarding the presence of NAFLD. NAFLD was seen three times as often in overweight/obese patients than in lean patients. Similarly, Borruel et al.^26^ found no difference in the prevalence of NAFLD in healthy women and those with PCOS; however, steatosis was eight times more prevalent in obese subjects. Interestingly, in our study the presence of NAFLD in the PCOS patients was significantly higher than in the controls for the lean participants. The comparisons of the PCOS and control groups drawn from the overweight/obese participants revealed no difference regarding the presence of NAFLD. Similarly, for all body weights, no difference regarding the presence of NAFLD was observed in the PCOS and control groups. These results can be explained as follows: The overweight/obese controls were at risk for NAFLD because of obesity. The overweight/obese patients with PCOS developed NAFLD by factors related to obesity and a deniable effect of PCOS. The lean controls were not prone to any known factor that could lead to NAFLD; however, the lean PCOS patients developed three times more NAFLD than controls and, had only PCOS as a known risk factor for NAFLD (Table 3). The significantly higher prevalence of NAFLD in the lean PCOS patients compared to the lean controls indicates that NAFLD is associated with PCOS independent of obesity and the related metabolic factors. In contrast, in the overweight/obese group, there was no difference in the prevalence of NAFLD in the PCOS group and the controls (Table 3). A meta-analysis by Ramezani-Binabaj et al.^5^ found a four-fold increase in the risk for NAFLD among women with PCOS, a result compatible only with the result of our lean patients, shows also an direct association of NAFLD and PCOS. The above mentioned study found that the prevalence of NAFLD in women with PCOS varied by country and that there was a relationship with genetics, ethnicity, and, especially, lifestyle, such as eating and exercise habits. It is possible that this characteristics of our study population resulted into an insignificant difference of NAFLD, between overweight/obese PCOS population and controls.

In the comparison of all of the PCOS patients and all of the controls, no significant difference in the prevalence of NAFLD was noted. The hypothetical direct effect of PCOS in the development of NAFLD was only present in the lean patients. This result could be related to the androgen levels, which have been reported to be associated with NAFLD in PCOS independent of obesity^27^, ^28^. Similarly, in a small-sized study, young lean women with PCOS were associated with insulin resistance but not with NAFLD; however, regular follow-ups were advised because of the known relationship between insulin resistance and NAFLD^6^. We advise regular follow-up of NAFLD for lean patients with PCOS. In a general population sample of 11,613 participants, lean NAFLD was independently associated with younger age and female sex. It was concluded that lean individuals with NAFLD have a different clinical profile than overweight/obese individuals with NAFLD^29^. For lean women, the association of PCOS and NAFLD and the related clinical parameters should be evaluated in further studies.

The results for studies on the relationship of NAFLD with BMI, waist circumference, metabolic syndrome, hyperandrogenism, and insulin resistance in patients with PCOS are conflicting^14^, ^27^, ^28^, ^30^. These conflicting results might be related to differences in population and study size. In the current study, of the patients with PCOS, significantly higher BMI, triglyceride levels, waist circumference, and FLI were found in those with NAFLD than in those without NAFLD.

The predictivity of the FLI for NAFLD in patients with PCOS was investigated with USG. Among all of the study population, the FLI was significantly higher in those with NAFLD than in those without NAFLD.

Despite a significant number of false negatives for the lean patients, the FLI was significantly higher for the PCOS patients with NAFLD than for the PCOS patients without NAFLD among all body weights (Table 2). This indirectly shows the power of FLI in the determination of NAFLD in overweight/obese PCOS patients.

The literature did not indicate whether NAFLD can be attributed to obesity or PCOS. In our study BMI had the strongest relationship with FLI. This might be another explanation for the failure of a finding of FLI as being predictive of NAFLD in lean patients.

All of the lean PCOS patients in this study had an FLI < 30. Similarly, Lerchbaum et al.^1^ observed no one with an FLI > 60 among lean patients with PCOS but its association with NAFLD was not investigated. Among our lean study population (FLI<30), NAFLD prevalence was significantly higher in the PCOS group than control females. This indicates that FLI < 30 could not rule out NAFLD in the lean PCOS patients. In this study we calculated a new cut-off value, 5.68, for FLI for the prediction of USG-confirmed NAFLD in lean PCOS and control patients. The use of FLI in lean PCOS patients with lower thresholds must be evaluated with further studies.

The limitations of this study include the lack of biopsy confirmation for NAFLD diagnosis; however, the accuracy of USG has been reported to be very high very high.^15^ A large majority of the studies in this field have adopted USG for NAFLD diagnoses because it is non-invasive. A notable and important strength of this study was the implementation of a very strict inclusion/exclusion protocol, which prevented the inclusion of patients with characteristics that could influence the results. This study is the first confirmation of NAFLD with USG for the evaluation of the predictivity of FLI in PCOS among different BMIs. This has contributed to the evaluation of a lean group without the effects of metabolic changes resulting from body composition.

The current study found that GGT activity was higher in women with PCOS. The FLI was highly correlated with BMI, followed by waist circumference and NAFLD. The FLI was correlated with NAFLD in the entire population. An FLI < 30 was insufficient for ruling out NAFLD in lean women with PCOS. For lean PCOS patients and controls a FLI value of 5.68 was the threshold for predicting the prescence or abscence of NAFLD. NAFLD and PCOS were associated, regardless of the metabolic factors related to obesity.

## Conclusion

FLI is highly correlated with BMI in patients with PCOS. In lean PCOS patiens a FLI<30 failed to rule out NAFLD.
